# Supplementary material for: Complexome profiling of the Chlamydomonas psb28 mutant reveals TEF5 as an early PSII assembly factor
Source: Plant Cell. 2025 Mar 17;37(6):koaf055. doi: 10.1093/plcell/koaf055 (PMC12164590; doi:10.1093/plcell/koaf055)
Supplement: koaf055_Supplementary_Data [file koaf055_supplementary_data.zip › psb28_interactive.zip!/plots/intensity/qualityPlots/peptides/PGM1_Cre06.g272050.t1.2.html]

### Cre06.g272050.t1.2

Trivial name: PGM1  
SeqCoverage [median]: 22.40  
Mass: 66.65 kDa  
  
 

| Sequence | MissedCleavage | Mass [Da] | wtIntensity | muIntensity | Score |
| --- | --- | --- | --- | --- | --- |
| AFEYEDGFTAFER | 0 | 1580.68 | 3084.39 | 0.00 | 127.42 |
| APHYFKDAK | 1 | 1075.55 | 521.30 | 0.00 | 52.59 |
| AVGLPSDADMGNSEVGHNALGSGQVVDQGAR | 0 | 3007.41 | 1776.49 | 1498.58 | 83.79 |
| DVPDGSSIR | 0 | 944.46 | 0.00 | 456.33 | 74.13 |
| EITAAGIEALK | 0 | 1114.62 | 6004.00 | 5337.20 | 74.92 |
| EITAAGIEALKSGK | 1 | 1386.77 | 1075.39 | 0.00 | 45.79 |
| FVGMMQYDGDLK | 0 | 1402.63 | 473.49 | 0.00 | 52.49 |
| INYANPDMVGHTGDMAATVR | 0 | 2131.98 | 7160.11 | 4568.22 | 79.97 |
| LVDLALETGR | 0 | 1085.61 | 11096.81 | 5058.19 | 94.69 |
| SGYLDAK | 0 | 752.37 | 1554.95 | 450.03 | 95.35 |
| TAVTTLR | 0 | 760.44 | 8411.96 | 5143.46 | 94.41 |
| TPTVDALR | 0 | 871.48 | 8109.81 | 2345.74 | 113.14 |
|  |  |  | 49268.7 | 24857.7 |  |
